# Supplementary material for: Low-Carbohydrate (Ketogenic) Diet in Children with Obesity: Part 1—Diet Impact on Anthropometric Indicators and Indicators of Metabolic Syndrome and Insulin Resistance
Source: Diseases. 2025 Mar 25;13(4):94. doi: 10.3390/diseases13040094 (PMC12026416; doi:10.3390/diseases13040094)
Supplement: Supplementary file 1 [file diseases-13-00094-s001.zip › diseases-3426013-supplementary.pdf]

Supplementary data

Low-Carbohydrate (Ketogenic) Diet in Children with Obesity: Part 1 – Diet Impact on Anthropometric Indicators and Indicators of Metabolic Syndrome and Insulin Resistance

I. Diet impact on anthropometric indicators according to age and gender.

Tabl.S1 Age-sex distribution of weight before and after the diet

	Age group	Gender	Mean	Std. Error	95% Confidence Interval Lower Bound	95% Confidence Interval Upper Bound	*F, df, P*
Mean weight before the KD (kg)	8-10 y.	male	51.567	11.865	27.758	75.375	*F*=0.258, *df*=5, p>0.05
		female	55.900	9.190	37.458	74.342	
	11-15 y.	male	88.818	4.381	80.026	97.610	
		female	85.533	11.865	61.725	109.342	
	16-18 y.	male	106.410	6.499	93.369	119.451	
		female	98.553	5.306	87.906	109.201	
Mean weight after the KD (kg)	8-10 y.	male	44.300	11.865	20.491	68.109	*F*=0.625, *df*=5, p>0.05
		female	52.600	9.191	34.158	71.042	
	11-15 y.	male	83.723	4.381	74.931	92.515	
		female	75.933	11.865	52.124	99.742	
	16-18 y.	male	100.410	6.499	87.369	113.451	
		female	89.567	5.306	78.919	100.214	

In order to determine whether there was a difference in the average weight before and after the diet by gender and age groups, a two-factor analysis of variance was conducted, where no statistically significant interaction effect of gender and age groups on the average weight of the patients during the two periods was found.

Fig.S1 Change in weight (kg) and weight loss percentage (%) in different age groups

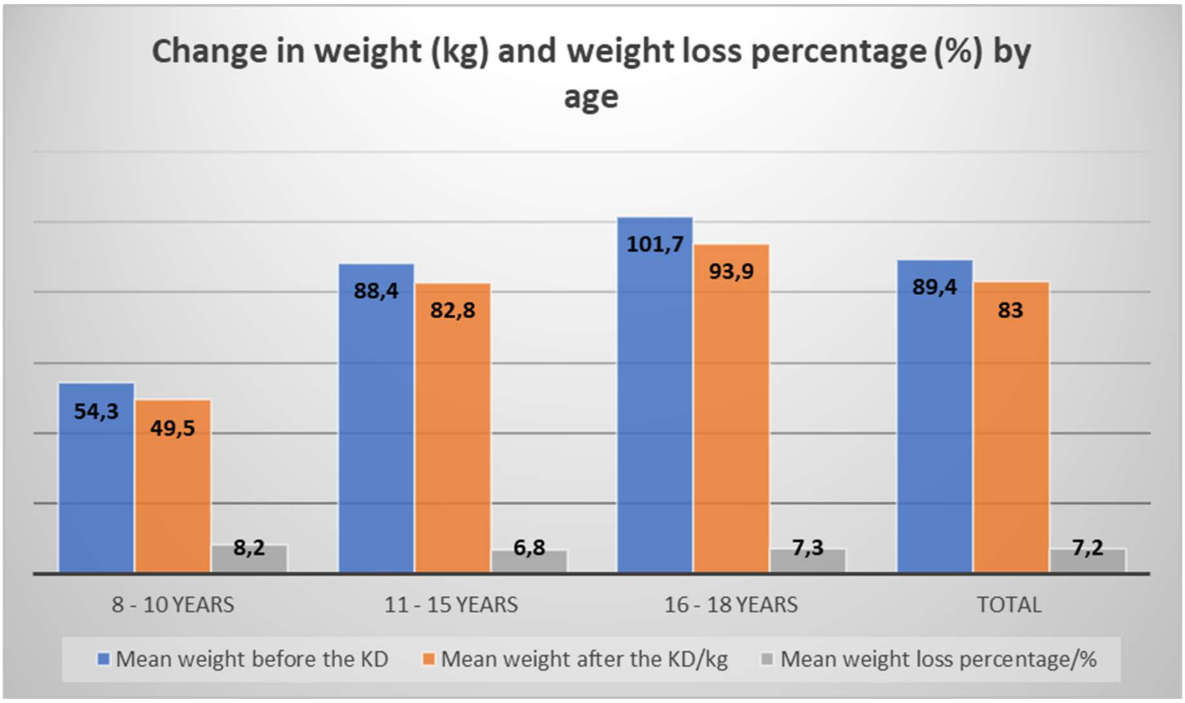

Tabl.S2 Comparison of BMI before and after the diet by gender

	Gender	Mean	Std. Deviation	N
BMI in kg/m2 before the KD	male	32.61943	5.585423	35
	female	34.45087	9.362830	23
	Total	33.34569	7.297953	58
BMI in kg/m2 after the KD	male	29.6340	5.46092	35
	female	31.1348	9.19766	23
	Total	30.2291	7.14060	58

The conducted analysis of variance revealed no statistically significant difference in the average BMI levels in kg/m² before and after completing the diet between boys and girls, $F(1, 56) = 2.46$, $p > 0.05$.

Tabl.S3 Comparison of BMI before and after the diet across age groups.

	Age group	Mean	Std. Deviation	N
BMI in kg/m2 before the KD	8-10 y.	26.72125	2.445430	8
	11-15 y.	32.86720	6.115126	25
	16-18y.	35.94400	8.090431	25
	Total	33.34569	7.297953	58
BMI in kg/m2 after the KD	8-10 y.	23.7625	2.42837	8
	11-15 y.	29.9600	6.18169	25
	16-18 y.	32.5676	7.83919	25
	Total	30.2291	7.14060	58

A decrease in BMI was observed in all age groups – specifically, -2.9 kg/m² in the first two age groups and -3.3 kg/m² in the third group of the oldest patients.

Fig.S2 BMI before and after the diet in different age groups

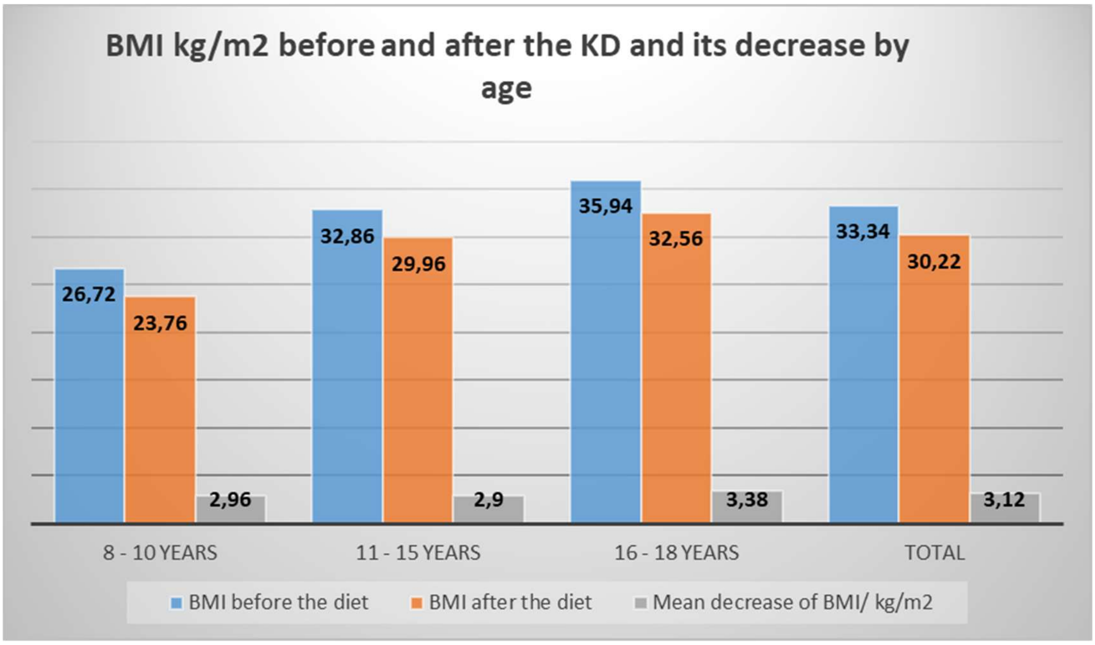

Tabl.S4 Waist-to-Height Ratio Comparison by Gender

	Gender	Mean	Std. Deviation	N
Waist-to-Height Ratio before the diet	Male	.6334	.06462	35
	female	.6457	.12738	23
	Total	.6383	.09375	58
Waist-to-Height Ratio after the diet	male	.5571	.06763	35
	female	.5417	.09466	23
	Total	.5510	.07902	58

The conducted analysis of variance did not reveal a statistically significant difference in the waist-to-height ratio by gender at both measurement stages, p>0.05.

Tabl.S5 Comparison of Waist-to-Height Ratio (WHR) by Age

	Age group	Mean	Std. Deviation	N
Waist-to-Height Ratio before the diet	8-10 y.	.6025	.04803	8
	11-15 y.	.6404	.07283	25
	16-18 y.	.6476	.11991	25
	Total	.6383	.09375	58
Waist-to-Height Ratio after the diet	8-10 y.	.5213	.03182	8
	11-15 y.	.5540	.08292	25
	16-18 y.	.5576	.08550	25
	Total	.5510	.07902	58

No statistically significant difference in the waist-to-height ratio was found among the three age groups at both measurement stages, p>0.05.

Fig.S3 Waist-to-Height ratio (WHR) before and after the diet in different age groups

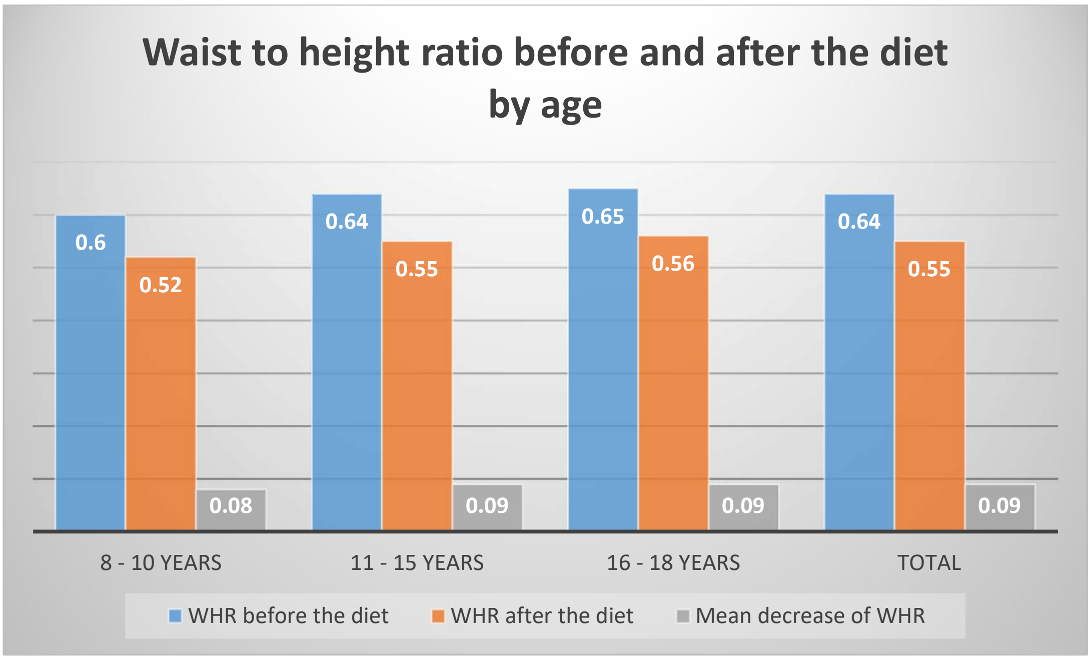

Tabl.S6 Average percentage of weight loss by gender

	N	Mean	Std. Deviation	Std. Error	95% Confidence Interval for Mean		Minimum	Maximum
					Lower Bound	Upper Bound		
male	35	-6.397	5.4742	.9253	-8.278	-4.517	-16.5	3.1
female	23	-8.391	4.8357	1.0083	-10.482	-6.300	-20.8	-.9
Total	58	-7.188	5.2791	.6932	-8.576	-5.800	-20.8	3.1

The average percentage of weight loss in the whole group was -7.18% (±0.69), with minimum values ranging from -20.8% to a maximum of 3.1%. A comparison by gender did not reveal a statistically significant difference in the mean values, $F(1, 56) = 2.02$, $p = 0.16$.

Tabl.S7 Average percentage of weight loss by age

	N	Mean	Std. Deviation	Std. Error	95% Confidence Interval for Mean		Minimum	Maximum
					Lower Bound	Upper Bound		
8-10 .y	8	-8.150	5.3899	1.9056	-12.656	-3.644	-16.5	-2.2
11-15 y.	25	-6.788	5.8748	1.1750	-9.213	-4.363	-20.8	2.4
16-18 y.	25	-7.280	4.7663	.9533	-9.247	-5.313	-14.5	3.1
Total	58	-7.188	5.2791	.6932	-8.576	-5.800	-20.8	3.1

The average percentage weight loss was highest in the youngest age group, but no statistically significant difference was found among the three age groups, $F(2, 55) = 0.203$, $p = 0.81$.

II. Diet impact on some laboratory indicators according to age and gender.

Tabl.S8 Fasting glucose before and after the KD by gender

Fasting glucose in mmol/l	N	Gender	Mean	SD	*F, df, P*
Glucose before the diet	35	boys	4.96	±0.40	F=0.24 df=1 p=0.62
	23	girls	4.90	±0.54	
Glucose after the diet	35	boys	4.92	±0.41	F=6.84 df=1 p=0.01
	23	girls	4.62	±0.42	

There is a statistically significant difference between boys and girls for the combined dependent variable: Wilk's Lambda = 0.94, $F(1) = 3.70$, $p = 0.03$. The mean values indicate that girls have a statistically significant decrease in fasting glucose.

Tabl.S9 Fasting glucose before and after the diet by age

Glucose mmol/l / Age group	N	Age group	Mean	SD	*F, df, P*
Glucose mmol/l before the diet	8	8-10 y.	4.85	±0.48	F=0.31
	25	11-15 y.	4.91	±0.39	df=2
	25	16-18 y.	4.98	±0.51	p=0.73
Glucose mmol/l after the diet	8	8-10 y.	4.71	±0.32	F=1.80
	25	11-15 y.	4.92	±0.41	df=2
	25	16-18 y.	4.71	±0.47	p=0.17

Tabl.S10 Fasting insulin before and after the KD by gender

Fasting insulin in mlU/l	N	Gender	Mean	SD	*F, df, P*
Fasting insulin in mlU/l before the diet	35	boys	20.45	±7.17	F=0.11 df=1 p=0.73
	23	girls	19.62	±11.16	
Fasting insulin in mlU/l after the diet	35	boys	14.57	±5.72	F=5.49 df=1 p=0.02
	23	girls	11.05	±5.41	

A statistically significant difference has been found between boys and girls for the combined dependent variable: Wilk's Lambda = 0.89, F(1) = 3.23, p = 0.04. The mean values indicate that girls have a statistically significant greater decrease in fasting insulin

Tabl.S11 Fasting insulin before and after the diet by age

Fasting insulin mlU/l / age group	N	Age grop	Mean	SD	*F, df, P*
Fasting insulin mlU/l before the diet	8	8-10 y.	14.39	±3.25	F=2.10
	25	11-15 y.	20.50	±7.69	df=2
	25	16-18 y.	21.58	±10.57	p=0.13
Fasting insulin mlU/l after the diet	8	8-10 y.	9.17	±1.96	F=2.51
	25	11-15 y.	14.32	±6.61	df=2
	25	16-18 y.	13.31	±5.37	p=0.09

Tabl.S12 HOMA-IR index before and after the KD by gender

HOMA-IR index/gender	N	Gender	Mean	SD	*F, df, P*
HOMA-IR index before the diet	35	boys	4.53	±1.67	F=0.01 df=1 p=0.91
	23	girls	4.47	±2.87	
HOMA-IR index after the diet	35	boys	3.21	±1.45	F=5.77 df=1 p=0.02
	23	girls	2.31	±1.30	

In the conducted one-way analysis of variance, no statistically significant difference was found in the mean levels of the HOMA-IR index between boys and girls at the beginning of the diet, $p > 0.05$. However, the mean level of the HOMA-IR index in girls is significantly lower than the measured value in boys after the completion of the diet.

Tabl.S13 HOMA-IR index before and after the diet in different age groups

HOMA-IR index/ age group	N	Age group	Mean	SD	*F, df, P*
HOMA-IR index before the diet	8	8-10 y.	3.15	±0.98	F=1.98
	25	11-15 y.	4.55	±1.77	df=2
	25	16-18 y.	4.90	±2.70	p=0.15
HOMA-IR index after the diet	8	8-10 y.	1.91	±0.43	F=2.41
	25	11-15 y.	3.17	1.65	df=2
	25	16-18 y.	2.82	1.34	p=0.10

Fig.S4 Distribution of the male patients according to the compliance with the diet %

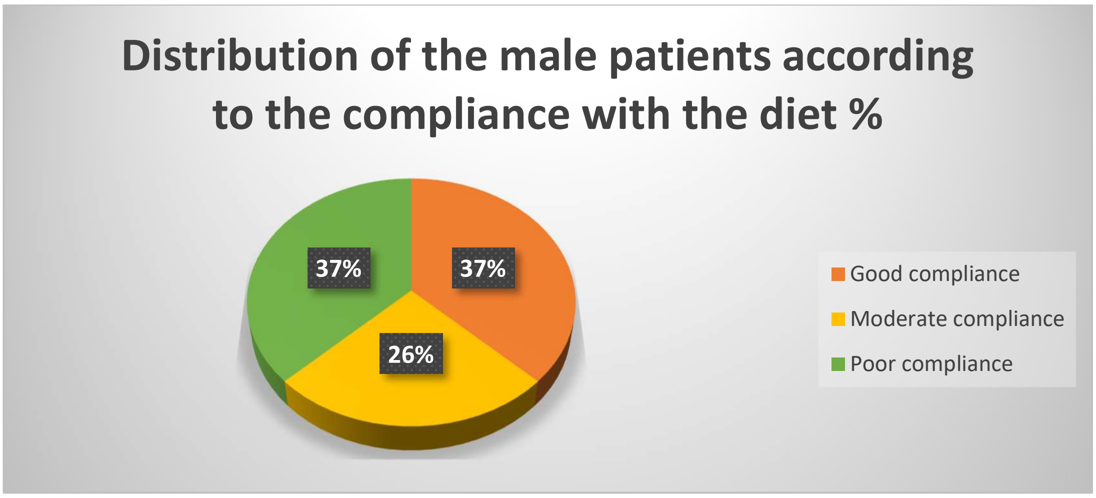

Fig.S5 Distribution of the female patients according to the compliance with the diet %

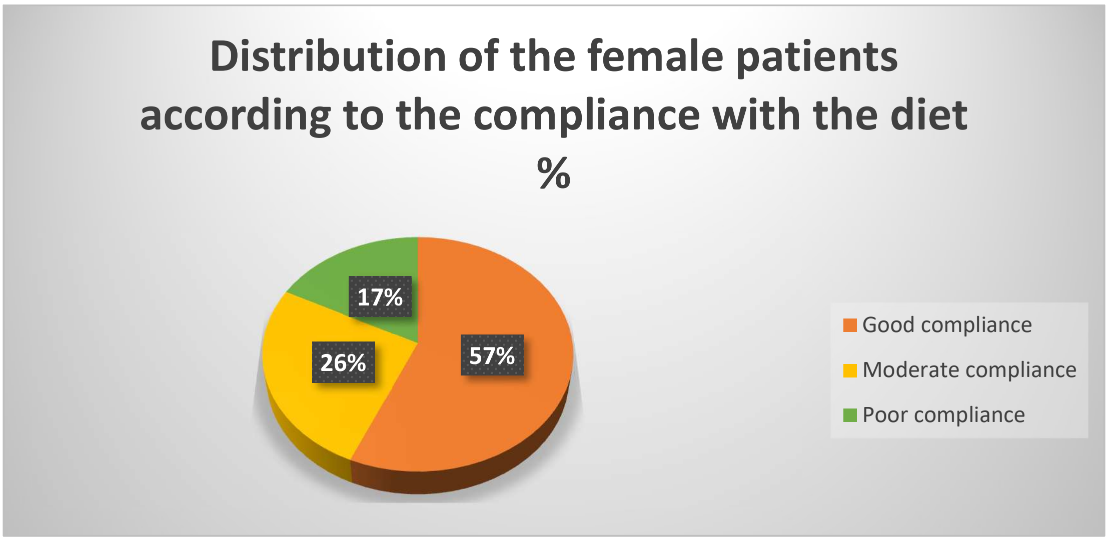

In the girls's group, patients with good compliance with the diet predominated, while in boys, the number of patients with good and poor compliance was equal.
